# Supplementary material for: Multi‐dataset identification of innovative feature genes and molecular mechanisms in keratoconus
Source: J Cell Mol Med. 2024 Sep 19;28(18):e70079. doi: 10.1111/jcmm.70079 (PMC11412914; doi:10.1111/jcmm.70079)
Supplement: Supplementary file 2 — Material S2. [file JCMM-28-e70079-s002.zip › 7.GSEA analysis/2.ATOH7/KEGG_AMINOACYL_TRNA_BIOSYNTHESIS.html]

Details for gene set KEGG\_AMINOACYL\_TRNA\_BIOSYNTHESIS[GSEA]

|  || Dataset | ATOH7.group.cls#h\_versus\_l |
| Phenotype | group.cls#h\_versus\_l |
| Upregulated in class | l |
| GeneSet | KEGG\_AMINOACYL\_TRNA\_BIOSYNTHESIS |
| Enrichment Score (ES) | -0.25597143 |
| Normalized Enrichment Score (NES) | -0.7926137 |
| Nominal p-value | 0.7427386 |
| FDR q-value | 1.0 |
| FWER p-Value | 1.0 |
Table: GSEA Results Summary

  

Fig 1: Enrichment plot: KEGG\_AMINOACYL\_TRNA\_BIOSYNTHESIS      
 Profile of the Running ES Score & Positions of GeneSet Members on the Rank Ordered List

  

| SYMBOL | TITLE | RANK IN GENE LIST | RANK METRIC SCORE | RUNNING ES | CORE ENRICHMENT || 1 | VARS2 | na | 8884 | 0.101 | -0.1317 | Yes |
| 2 | DARS2 | na | 10206 | 0.078 | -0.0739 | Yes |
| 3 | YARS2 | na | 11237 | 0.063 | -0.0267 | Yes |
| 4 | HARS2 | na | 11258 | 0.062 | 0.0494 | Yes |
| 5 | FARSA | na | 13724 | 0.033 | 0.0191 | Yes |
| 6 | FARS2 | na | 13845 | 0.032 | 0.0550 | Yes |
| 7 | RARS2 | na | 14013 | 0.030 | 0.0876 | Yes |
| 8 | SARS2 | na | 14024 | 0.030 | 0.1245 | Yes |
| 9 | PSTK | na | 14346 | 0.027 | 0.1486 | Yes |
| 10 | MTFMT | na | 15301 | 0.018 | 0.1438 | Yes |
| 11 | NARS2 | na | 15832 | 0.014 | 0.1459 | Yes |
| 12 | FARSB | na | 16136 | 0.012 | 0.1517 | Yes |
| 13 | AARS2 | na | 16298 | 0.010 | 0.1599 | Yes |
| 14 | TARS2 | na | 16878 | 0.006 | 0.1501 | Yes |
| 15 | EARS2 | na | 17287 | 0.002 | 0.1407 | Yes |
| 16 | WARS2 | na | 17612 | -0.001 | 0.1322 | Yes |
| 17 | IARS2 | na | 18419 | -0.008 | 0.1185 | Yes |
| 18 | PARS2 | na | 20229 | -0.024 | 0.0958 | Yes |
| 19 | LARS2 | na | 21532 | -0.038 | 0.1044 | Yes |
| 20 | SEPSECS | na | 22567 | -0.049 | 0.1350 | Yes |
| 21 | CARS2 | na | 22763 | -0.052 | 0.1928 | Yes |
| 22 | MARS2 | na | 26935 | -0.124 | 0.2245 | Yes |
Table: GSEA details [plain text format]

  

Fig 2: KEGG\_AMINOACYL\_TRNA\_BIOSYNTHESIS      
 Blue-Pink O' Gram in the Space of the Analyzed GeneSet

  

Fig 3: KEGG\_AMINOACYL\_TRNA\_BIOSYNTHESIS: Random ES distribution      
 Gene set null distribution of ES for **KEGG\_AMINOACYL\_TRNA\_BIOSYNTHESIS**
